# Unusual Dermoid Cyst in Oral Cavity

**DOI:** 10.1155/2014/389752

**Published:** 2014-04-10

**Authors:** Evanice Menezes Marçal Vieira, Alvaro Henrique Borges, Luis Evaristo Ricci Volpato, Alessandra Nogueira Porto, Artur Aburad Carvalhosa, Gilberto de Almeida Botelho, Matheus Coelho Bandeca

**Affiliations:** ^1^Department of Post-Graduation in Integrated Dental Sciences, University of Cuiabá, Avenida Beira Rio 3100, 78025-190 Cuiabá, MT, Brazil; ^2^Post-Graduate Program in Dentistry, CEUMA University, 01 Josue Montello, 65075-120 São Luis, MA, Brazil

## Abstract

Dermoid cysts in oral cavity are unusual lesions. Their etiology is not yet clear and can be associated with trapped cells as a result of the inclusion error resulting in the development into the ectoderm, mesoderm, and endoderm tissues. The aim of this case report is to evidence the presence of a dermoid cyst in the floor of mouth surgically removed. In the present case, the lesion showed soft consistency, floating, regular borders, smooth surface, and the same color as the adjacent mucosa, asymptomatic and measuring 4.5 × 5.5 cm in its greatest diameter. The initial diagnostic was ranula in consequence of the similarity with clinical characteristics and localization. After surgical removal lesion, a fibrotic capsule was identified with a friable material with intensive yellow color. The microscopic exam showed cystic lesion with cavity lined by squamous stratified epithelium hyperorthokeratinized. Cutaneous attachments, such as sebaceous glands and hair follicles, were present in connective adjacent tissue. Surgical intervention is elective in these situations. All dentists must have a thorough knowledge of this unusual lesion.

## 1. Introduction

Dermoid cysts are developmental lesions that arise either by entrapped pluripotent cells or by implantation of epithelium, with the former being termed congenital and the latter as acquired [1–3]. Nowadays, the etiology of dermoid cysts is not yet clear and can be associated with trapped cells as a result of the inclusion error resulting in the development into the ectoderm, mesoderm, and endoderm tissues [4]. These conditions may produce hair, muscle, bone, cartilage, teeth, and mucous membranes. Historical trauma, infection, and spontaneous autonomous new growth are closely related to these lesions [4].

Considering the histological aspects, the lesions are classified as epidermoid cyst (lined by only by stratified squamous epithelium and composed by ectodermic layer), dermoid cyst (lined by stratified epithelium with skin adnexa), and teratoid (can be cystic or solid featured other tissues such as muscle, cartilage, or bone are present) [4, 5]. These benign lesions are encountered throughout the body and rarely occur in the head and neck region, 1.6 to 7%, and represent less than 0.01% of all oral cavity cysts [2, 6]. There is no sex predilection and the dermoid cysts are common affecting people between the ages of 15 and 35 years [7]. Also, they are frequently found in sites where embryonic parts fuse together. The majority of reported cases are in the midline of the body, as well as in the ovaries, and in the testicles.

Usually these lesions are asymptomatic; however, their slow enlargement can cause obstruction with consequent dysphagia, dysphonia, and at last dyspnea. The size of dermoid cysts is very variable (up to ten cm in diameter) and it depends on their first clinic manifestation [6, 8]. The treatment of choice is surgical excision [9]. Recurrence of the lesion is unusual [10]. The aim of this case report is to evidence the presence of a dermoid cyst in the floor of mouth surgically removed.

## 2. Case Report

**Figure 1:**
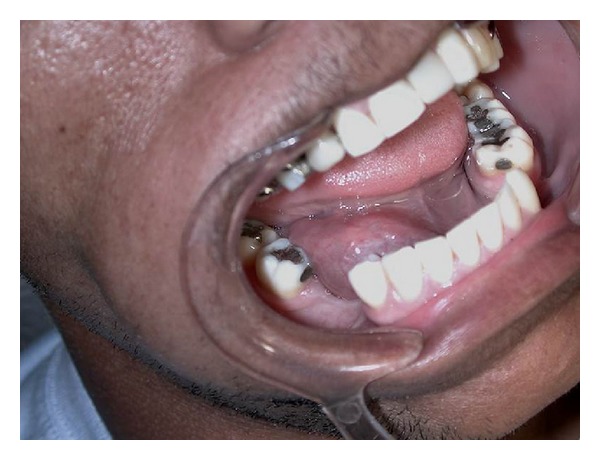
Clinical view of the dermoid cyst showing soft consistency, floating, regular borders, smooth surface, and the same color as the adjacent mucosa.

A 29-year-old black man reported to the Semiology Clinic at Dental School of the University of Cuiabá, Cuiabá-MT. During the intrabuccal exam, the dentist noted a volumetric increase with slow growth in the floor of the mouth, laterally to tongue. The lesion showed soft consistency, floating, regular borders, smooth surface, and the same color as the adjacent mucosa, asymptomatic and measuring 4.5 × 5.5 cm in its greatest diameter (Figure 1). The patient also presented dysphonia to speak some words.

**Figure 2:**
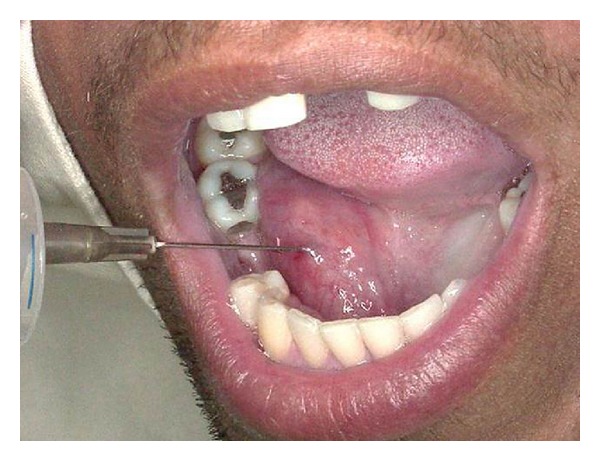
An aspiration puncture with a thick needle was done and no material was collected.

Before the biopsy, the suspected diagnosis was ranula due the similarity to clinical characteristics and localization. An aspiration puncture with a thick needle was done and no material was collected. Based on this, the possibility of a solid or a cystic lesion with semisolid container was considered (Figure 2).

**Figure 3:**
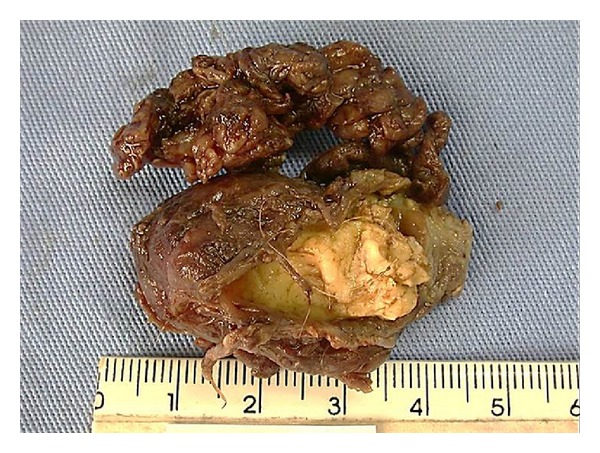
The lesion was completely removed and submitted to biopsy.

**Figure 4:**
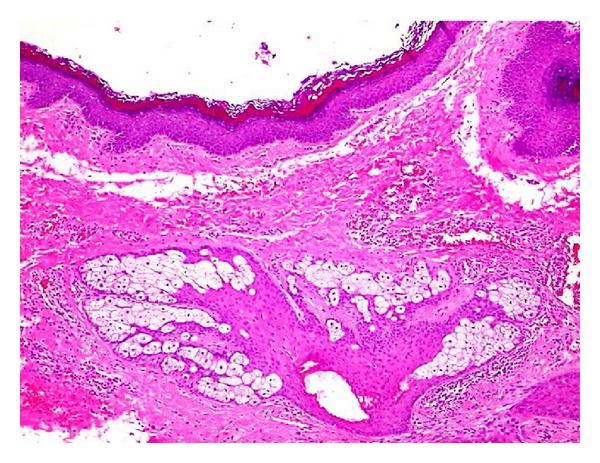
The microscopic exam showed a cystic lesion with cavity lined by squamous stratified epithelium hyperorthokeratinized with cutaneous attachments, such as sebaceous glands and hair follicles.

The treatment administered in the present case was surgical and the lesion was completely removed (Figure 3). Initially, the patient was examined and no systemic involvement was observed. The collected material was followed to be analyzed in the Surgical Pathology Laboratory of the University of Cuiabá. Covering the lesion, a fibrotic capsule was identified with a friable material with intensive yellow color. The microscopic exam showed cystic lesion with cavity lined by squamous stratified epithelium hyperorthokeratinized. Cutaneous attachments, such as sebaceous glands and hair follicles, were present in connective adjacent tissue (Figure 4).

## 3. Discussion

When found in oral cavity, dermoid cysts are classified as nonodontogenic lesions and about 7% occur in head/neck region, among them 23% are located at floor of mouth and can be found either lateral to tongue or in the midline [11, 12]. They are caused by the retention of the germinal epithelium during the growth of the mandible and hyoid branchial arches [11]. Even though they are generally diagnosed in the second and third decades of life, they can present at any age with equal frequency of occurrence to both genders [7]. Depending on the size of the lesion, it can displace the tongue and cause dysphagia, dysphonia, and dyspnea [13]. This case report presented a dermoid cyst at the floor of mouth in a 29-year-old male patient that sought for care reporting difficulties to pronounce some words.

The differential diagnosis of lesions that present as a cyst or pseudocyst of the floor of the mouth includes mucocele, ranula, cystic hygroma, thyroglossal duct cyst, brachial cleft cyst, infectious process, lymphatic malformation, tumors, hemangioma, salivary lesions, and Ludwig's angina only in cases of inflammatory complications [14]. The clinical evaluation of the lesion is asymptomatic and may present as slow growing. The size of the cyst is variable from millimeters till some centimeters, depending on its first clinical manifestation [6]. Even the aspiration biopsy is commonly used; in many cases, it can result in a not reliable diagnostic sample [7]. Histologically, dermoid cysts are lined by epidermis with the contents of keratinaceous, caseous, sebaceous, or purulent with hair, nails, fat globules, and even cartilage [2, 4].

The treatment of choice is surgical enucleation via an intraoral or extraoral approach, which is facilitated by the presence of a capsule [15]. An intraoral approach is recommended in cases of cysts above the mylohyoid muscle and the extraoral technique is chosen in very large lesions which affect submandibular region and in situations of infection process that may interfere to patient's airway [2]. In present case, the cyst presented the extension of 5.5 cm and then an intraoral approach was preferred to lead to cosmetic and functional results [2, 3]. The tax of recurrence is low when the nucleation of the fibrous capsule of these lesions were made, but it should be considered the possibility of malignant transformation of oral dermoid cysts into the teratoid type [16].

## 4. Conclusion

Based on the case report, it was possible to observe the importance of differential diagnosis in relation to other nodule mass lesions and that the histological aspects are conclusive to define the treatment. Surgical intervention is elective in these situations.
